# Assessing quality of hepato-pancreato-biliary surgery: nationwide benchmarking

**DOI:** 10.1093/bjs/znae119

**Published:** 2024-05-15

**Authors:** Michelle R de Graaff, Tessa E Hendriks, Michel Wouters, Mark Nielen, Ignace de Hingh, Bas Groot Koerkamp, Hjalmar C van Santvoort, Olivier R Busch, Marcel den Dulk, Joost M Klaase, Erik van Zwet, Bert A Bonsing, Dirk J Grünhagen, Marc G Besselink, Niels F M Kok

**Affiliations:** Dutch Institute for Clinical Auditing, Scientific Bureau, Leiden, The Netherlands; Department of Hepato-Pancreato-Biliary Surgery and Liver Transplantation, University Medical Centre Groningen, Groningen, the Netherlands; Dutch Institute for Clinical Auditing, Scientific Bureau, Leiden, The Netherlands; Amsterdam UMC, location University of Amsterdam, Department of Surgery, Amsterdam, The Netherlands; Department of Surgery, Cancer Centre Amsterdam, Amsterdam, The Netherlands; Department of Surgery, Leiden University Medical Centre, Leiden, The Netherlands; Dutch Institute for Clinical Auditing, Scientific Bureau, Leiden, The Netherlands; Department of Surgical Oncology, Netherlands Cancer Institute, Amsterdam, The Netherlands; Department of Biomedical Data Sciences, Leiden University Medical Centre, Leiden, The Netherlands; Dutch Institute for Clinical Auditing, Scientific Bureau, Leiden, The Netherlands; Department of Surgery, Catharina Hospital, Eindhoven, The Netherlands; Department of Surgery, Erasmus MC Cancer Institute, Rotterdam, The Netherlands; Department of Surgery, University Medical Centre Utrecht, Utrecht, The Netherlands; Department of Surgery, St Antonius Hospital, Nieuwegein, the Netherlands; Amsterdam UMC, location University of Amsterdam, Department of Surgery, Amsterdam, The Netherlands; Department of Surgery, Cancer Centre Amsterdam, Amsterdam, The Netherlands; Department of Surgery, Maastricht University Medical Centre, Maastricht, The Netherlands; NUTRIM-School of Nutrition and Translational Research in Metabolism, Maastricht University, Maastricht, The Netherlands; Department of Hepato-Pancreato-Biliary Surgery and Liver Transplantation, University Medical Centre Groningen, Groningen, the Netherlands; Department of Biomedical Data Sciences, Leiden University Medical Centre, Leiden, The Netherlands; Department of Surgery, Leiden University Medical Centre, Leiden, The Netherlands; Department of Surgery, Erasmus MC Cancer Institute, Rotterdam, The Netherlands; Amsterdam UMC, location University of Amsterdam, Department of Surgery, Amsterdam, The Netherlands; Department of Surgery, Cancer Centre Amsterdam, Amsterdam, The Netherlands; Department of Surgical Oncology, Netherlands Cancer Institute, Amsterdam, The Netherlands

## Abstract

**Background:**

Clinical auditing is a powerful tool to evaluate and improve healthcare. Deviations from the expected quality of care are identified by benchmarking the results of individual hospitals using national averages. This study aimed to evaluate the use of quality indicators for benchmarking hepato-pancreato-biliary (HPB) surgery and when outlier hospitals could be identified.

**Methods:**

A population-based study used data from two nationwide Dutch HPB audits (DHBA and DPCA) from 2014 to 2021. Sample size calculations determined the threshold (in percentage points) to identify centres as statistical outliers, based on current volume requirements (annual minimum of 20 resections) on a two-year period (2020–2021), covering mortality rate, failure to rescue (FTR), major morbidity rate and textbook/ideal outcome (TO) for minor liver resection (LR), major LR, pancreaticoduodenectomy (PD) and distal pancreatectomy (DP).

**Results:**

In total, 10 963 and 7365 patients who underwent liver and pancreatic resection respectively were included. Benchmark and corresponding range of mortality rates were 0.6% (0 −3.2%) and 3.3% (0–16.7%) for minor and major LR, and 2.7% (0–7.0%) and 0.6% (0–4.2%) for PD and DP respectively. FTR rates were 5.4% (0–33.3%), 14.2% (0–100%), 7.5% (1.6%–28.5%) and 3.1% (0–14.9%). For major morbidity rate, corresponding rates were 9.8% (0–20.5%), 28.1% (0–47.1%), 36% (15.8%–58.3%) and 22.3% (5.2%–46.1%). For TO, corresponding rates were 73.6% (61.3%–94.4%), 54.1% (35.3–100), 46.8% (25.3%–59.4%) and 63.3% (30.7%–84.6%). Mortality rate thresholds indicating a significant outlier were 8.6% and 15.4% for minor and major LR and 14.2% and 8.6% for PD and DP. For FTR, these thresholds were 17.9%, 31.6%, 22.9% and 15.0%. For major morbidity rate, these thresholds were 26.1%, 49.7%, 57.9% and 52.9% respectively. For TO, lower thresholds were 52.5%, 32.5%, 25.8% and 41.4% respectively. Higher hospital volumes decrease thresholds to detect outliers.

**Conclusion:**

Current event rates and minimum volume requirements per hospital are too low to detect any meaningful between hospital differences in mortality rate and FTR. Major morbidity rate and TO are better candidates to use for benchmarking.

## Teaser

This nationwide audit-based study exposed the complexity of benchmarking quality of care in complex, relatively low frequent surgical procedures in HPB surgery. Current event rates and minimum volume requirements per hospital are too low to detect any meaningful between hospital differences in mortality rate and failure to rescue. Major morbidity rate and textbook or ideal outcome are better candidates to use for benchmarking, as the event rates of these quality indicators are higher.

## Introduction

Clinical auditing is a powerful tool to evaluate and improve healthcare.^1,2^ Data are collected to measure structure, processes, and care outcomes using surgical quality indicators (QIs). Deviations from the expected quality of care are identified by benchmarking the results of individual hospitals using national averages.

Transparency and accountability within healthcare is increasingly demanded. Obligatory and online accessible hospital data on quality measures reflect this^3^. For benchmarking hospital performance using publicly available QIs, it is important to ensure these indicators are accurate and relevant to maintain their effectiveness. Hospital differences in QIs are often displayed using funnel plots. These graphs show observed outcome rates plotted against the population benchmark and contain 95% prediction intervals around the benchmark. Hospitals that deviate from these intervals are considered potential outliers. However the probability of detecting an outlier hospital using these funnel plots depends on hospital volume and average event rate of all hospitals.

In the Netherlands, there is a minimum required annual volume of 20 liver resections (LR) and 20 pancreatoduodenectomies per centre. When volumes are small, it is not possible to distinguish clinically relevant differences in QIs from differences due to statistical uncertainty. This is particularly true for QIs with a low event rate such as postoperative mortality rate^4,5^. Consequently, several quality measures may not have enough discriminating power in the current system to distinguish lower quality from statistical variation.

In 2022, the Dutch government presented an ambitious multiparty healthcare agreement, aiming to enhance healthcare quality and transparency of healthcare services. One key aspect of this agreement is centralization of complex and high-risk surgical procedures^6^. A new minimum annual volume between 50 and 100 resections per hospital is proposed^7^. Evidence for the increase of minimal hospital volume is conflicting, however. Discussions on centralization of complex surgical procedures are ongoing in several healthcare systems. This study aimed to evaluate whether four different QIs could be used to benchmark quality of hepato-pancreato-biliary (HPB) surgery and under what condition outlier hospitals could be identified.

## Methods

This study was conducted with data from the Dutch Hepatobiliary Audit (DHBA) and the Dutch Pancreatic Cancer Audit (DPCA), the mandatory clinical audits for liver and pancreatic surgery in the Netherlands. Since 2014, all liver and pancreatic resections in the Netherlands have been included in the DHBA and DPCA. Previous data verification of the DHBA and DPCA showed estimated data completeness exceeding 97%^8,9^. Both audits are managed by the Dutch Institute of Clinical Auditing (DICA), founded in 2010. DICA facilitates clinical auditing using a validated systematic analysis of the quality of care. DICA advocates the use of funnel plots to show hospital differences.

Since 2011, some centralization of liver and pancreatic surgery has taken place in the Netherlands. This resulted in fewer hospitals performing liver and pancreatic surgery. From 2014 to 2021, the number of centres performing liver surgery decreased from 27 to 21 and those performing pancreatic surgery from 22 to 15. National requirements to perform liver surgery include a minimal annual hospital volume of 20 liver resections, experienced staff, and access to other local therapies, including thermal ablation and radiation^10^. National requirements to perform pancreatic surgery include a minimal annual number of 20 pancreatoduodenectomies (PD). There are currently no volume requirements for distal pancreatectomy (DP)^11^. The scientific committees of the DHBA and DPCA approved this study protocol^12^. According to Dutch law, no ethical approval or informed consent was needed because all data are registered de-identified.

### Patient selection

This study included data of patients who underwent liver resection and patients who underwent pancreatoduodenectomy and distal pancreatectomy according to the inclusion criteria of the respective national audits. All patients registered in the DHBA or DPCA between 1 January 2014 and 31 December 2021 were eligible.

Exclusion criteria were patients treated with thermal liver ablation alone, or patients who underwent total pancreatectomy or central pancreatectomy.

### Treatment groups

Patients who undergo liver or pancreatic resections form a heterogeneous group due to tumour location, extent of resection and wide variation in patient and tumour characteristics for distinct tumour types. For liver resection, therefore, treatment groups were divided according to tumour type (colorectal liver metastases (CRLM), hepatocellular carcinoma (HCC), perihilar cholangiocarcinoma (pCCA) and intrahepatic cholangiocarcinoma (iCCA)) and extent of liver surgery (major and minor LR). Major LR was defined as three or more adjacent liver segments. Pancreatic resections were divided into pancreatoduodenectomy (all types of pancreatoduodenectomy: pylorus resecting, pylorus preserving, and ‘classical’ Whipple) and distal pancreatectomy (that is, left-sided pancreatectomy, with or without the pancreatic body and spleen).

### Outcomes

The main outcome was the absolute percent point difference necessary (threshold) to identify a centre as a statistical outlier compared to the benchmark, considering current volume requirements (20 resections per year) and new proposed volume requirements (50–100 resections per year), measured over a one- and two-year period. These were calculated for four QIs including mortality rate, major morbidity rate, failure to rescue (FTR) and the composite endpoints textbook outcome (TO)^13^ for liver surgery and ideal outcome (IO)^14^ for pancreatic surgery.

#### Definitions

An outlier was defined as a hospital that fell outside 95% prediction intervals, indicating significantly worse performance compared to the benchmark. It was deliberately chosen not to look at differences in the context of better performance, as the aim of benchmarking in the context of the four QIs is primarily intended to reduce ‘negative’ differences.

Mortality rate was defined as death during initial hospital admission or within 30 days of surgery in case of earlier discharge. Major morbidity rate was defined as Clavien–Dindo grade ≥3a complication. Failure to rescue was defined as in-hospital or 30-day mortality rate after a Clavien–Dindo grade ≥3a complication. TO was achieved in the absence of severe postoperative complications, death, readmission, or prolonged length of stay (≤90th percentile) and when adequate surgical resection margins were obtained^13^. IO was defined as the combined absence of in-hospital mortality, severe complications (Clavien–Dindo ≥3a), postoperative pancreatic fistula (POPF) (grade B/C, defined in accordance with the International Study Group of Pancreatic Surgery (ISGPS) classification)^15^, reoperation while maintaining an acceptable postoperative length of stay (≤75th percentile) without readmission^14^.

### Statistical analysis

All analyses were performed using R version 4.1.0 (R Core Team (2021), R: A language and environment for statistical computing. R Foundation for Statistical Computing, Vienna, Austria).

The benchmark was calculated as the national mean event rate of mortality rate, major morbidity rate, FTR, and TO or IO over 2020–2021 for each treatment group. In addition, a series of sample size calculations was performed to determine the minimum volume needed to detect an outlier hospital. These calculations were based on a one-sample proportion test with a power (β) of 80% and a two-sided significance level (α) of 0.05. The hypothesis of a two-sided test states that the proportions in the two groups are unequal, either higher or lower. The equation is shown in *Fig. S1*.

Absolute numbers of patients who underwent a surgical procedure per treatment group per hospital were presented. The absolute percentage point differences necessary to identify a hospital as a statistical outlier compared to the benchmark for these four QIs were plotted against hospital volumes. Special attention was given to the ability to discriminate a hospital as a negative outlier when 20, 40, 50, 100 and 200 resections were performed. Then, a series of sample size calculations was deducted to assess the minimum surgical hospital volume needed to identify a significant outlier. As this is a sliding scale, specific calculations were performed for a rate that was two-fold of the benchmark and an absolute 2%-, 5%- and 15%-point increase in mortality rate, FTR and major morbidity rate as compared to the benchmark. Because an increase in TO or IO rates indicates a better-performing hospital, these calculations were based on a decrease in absolute percentage points compared to the benchmark. The proportion of hospitals exceeding the needed volume per treatment group was calculated by comparing a hospital’s actual volume met within 1, 2, 3, 5 and 8 years, with the necessary sample size to detect a statistically significant outlier hospital.

## Results

Overall, 18 328 HPB surgical procedures were included from 24 hospitals including 10 963 (59.8%) liver resections and 7365 (40.2%) pancreatic resections. During the study period, 12 hospitals performed both liver and pancreatic surgery, 2 hospitals performed only pancreatic surgery and 10 hospitals performed only liver surgery. Median hospital volumes per treatment group are shown in *Table 1*. For each QI the nationwide two-year benchmark was calculated and displayed in *Table S1*.

**Table 1:** Number of hospitals performing specific HPB procedures and nationwide average rates (benchmark) of quality indicators. All numbers are calculated with 2020 and 2021 data

	Liver resection		Pancreatic resections	
	Minor	Major	Pancreaticoduodenectomy	Distal pancreatectomy
Number of hospitals	21	21	15	15
Total number of procedures 2014–2021	8036	2761	5808	1557
Median number of procedures per hospital (min–max) in 2021	44 (9–84)	9 (1–54)	39 (22–144)	13 (5–30)
Nationwide average rate of mortality (min–max) in %	0.6 (0–3.2)	3.3 (0–16.7)	2.7 (0–7.0)	0.6 (0–4.2)
Nationwide average rate of major morbidity (min–max) in %	9.8 (0–20.5)	28.1 (0–47.1)	36. (15.8–58.3)	22.3 (5.2–46.1)
Nationwide average rate of failure to rescue (min–max) in %	5.4 (0–33.3)	14.2 (0–100)	7.5 (1.6–28.5)	3.1 (0–14.9)
Nationwide average rate of textbook outcome (min–max) in %	73.6 (61.3–94.4)	54.1 (35.3–100)	46.8 (25.3–59.4)	63.3 (30.7–84.6)

**Fig. 1:**
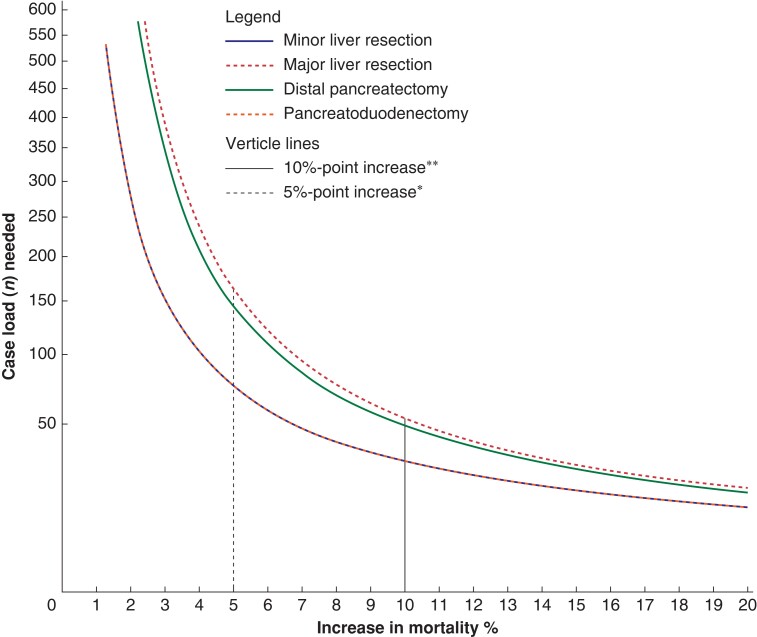
Required caseload per percentage point increase compared to the benchmark for minor and major liver resections and PD and distal pancreatectomies regarding mortality rate

In *Fig. 1* the minimum volume is plotted against absolute point increase of mortality to statistically detect a hospital with higher postoperative mortality. Higher volumes allow for distinguishing smaller percentage-point differences. *Figure S2* shows different required volumes for a gliding scale of absolute percentage point differences and *Tables S2–5* show the proportion of hospitals meeting or exceeding these volumes for different thresholds and for a varying number of registration years.

*To be able to distinguish a 5%-point increase compared to the benchmark for mortality rate would require a hospital volume of 75 minor liver resections, 164 major liver resections, 146 pancreaticoduodenectomies and 75 distal pancreatectomies respectively. **To be able to distinguish a 10%-point increase compared to the benchmark for mortality rate would require a hospital volume of 31 for minor liver resections, 54 major liver resections, 49 pancreaticoduodenectomies and 31 distal pancreatectomies respectively.

### Mortality rate

Benchmark and corresponding range of mortality rates were 0.6% (0–3.2%) and 3.3% (0–16.7%) for minor and major LR, and 2.7% (0–7.0%) and 0.6% (0–4.2%) for PD and DP respectively (*Table 1*).

An overview at which absolute percentage point increase a hospital could be identified as an outlier for different minimal hospital volumes is given in *Table 2*. Thresholds indicating a significant outlier for mortality with the current required minimal volume of 20, measured over a 2-year period, were 8.6%, 15.4%, 14.2% and 8.6% for minor LR, major LR, PD and DP respectively.

**Table 2:** The absolute percentage point increase necessary to identify a centre as a statistical outlier compared to the benchmark, considering current volume requirements (20 resections per year) and new proposed volume requirements (50–100 resections per year), measured over a one- and a two-year period

QI	Volume	Minor LR	Major LR	Pancreaticoduodenectomy	Distal pancreatectomy
		Absolute percent-point difference	Absolute percent-point difference	Absolute percent-point difference	Absolute percent-point difference
Mortality rate	20	13.9	19.3	18.6	13.9
	40	8.0	12.1	11.6	8.0
	50	6.8	10.4	10.0	6.8
	100	4.1	6.7	6.4	4.1
	200	2.5	4.4	4.2	2.5
FTR	20	19.8	26.2	23.5	19.0
	40	12.5	17.5	15.4	11.9
	50	10.8	15.4	13.5	10.3
	100	7.0	10.4	9.0	6.6
	200	4.6	7.1	6.0	4.4
Major morbidity rate	20	25.0	30.7	30.7	29.6
	40	16.6	21.6	21.9	20.5
	50	14.5	19.2	19.6	18.2
	100	9.7	13.5	13.8	12.6
	200	6.6	9.4	9.7	8.7
Textbook/ideal outcome	20	30.3	29.9	28.6	30.7
	40	21.1	21.6	21.1	21.9
	50	18.8	19.4	19.0	19.6
	100	13.1	13.8	13.6	13.8
	200	9.1	9.7	9.8	9.7

For example, in a scenario where all hospitals conduct a minimum of 40 minor liver resections in 2 years. Based on the current benchmark of mortality rate (0.6%), a hospital would be marked as outlier when the mortality rate of this hospital is 6.8%-points higher compared to the benchmark (7.4%). In a scenario in which a minimum of 100 minor liver resections would be performed, a hospital would be marked as underperforming when mortality rate is 4.1%-points higher compared to the benchmark (4.7%).

To statistically distinguish smaller differences such as a 5% absolute increase in mortality in three pooled years a minimum annual hospital volume of 25 minor LR, 54 major LR, 48 PDs and 25 DPs would be required, only for minor LR was this met by 80% of the hospitals (*Table S2*).

### Failure to rescue

Benchmark and corresponding range of FTR were 5.4% (0–33.3%), 14.2 (0–100%), 7.5% (1.6%–28.5%) and 3.1% (0–14.9%) for minor LR, major LR, PD and DP respectively. Thresholds indicating a significant outlier for FTR with the current required minimal volume of 20, measured over 2 years, were 17.9%, 31.6%, 22.9% and 15.0% respectively (*Table 1*).

When stakeholders would like to be able to distinguish an outlier with a 5% absolute increase to the benchmark, less than 25% of hospitals would currently meet the required volume in three successive years for all four types of surgery (*Table S3*).

### Major morbidity rate

Benchmark and corresponding range for major morbidity corresponding rates were 9.8% (0–20.5%), 28.1% (0–47.1%), 36% (15.8%–58.3%) and 22.3% (5.2%–46.1%) for minor LR, major LR, PD and DP respectively (*Table 1*). Thresholds indicating a significant outlier for mortality with the current required minimal volume of 20, measured over 2 years, were 26.1%, 49.7%, 57.9%, and 52.9% respectively.

The minimum volume needed to reliably distinguish a 5% absolute increase in major morbidity rate compared to the benchmark is not reached by any hospital when pooling data over 3 consecutive years (*Table S4*).

### Textbook outcome/ideal outcome

Benchmark and corresponding for TO were 73.6% (61.3%–94.4%, minor LR), 54.1% (35.3–100%, major LR), and for IO were 46.8% (25.3%–59.4%, PD) and 63.3% (30.7%–84.6%, DP); *Table 1*. Thresholds indicating a significant outlier for mortality with the current required minimal volume of 20, measured over 2 years, were 52.5%, 32.5%, 25.8% and 41.4% respectively.

**Fig. 2:**
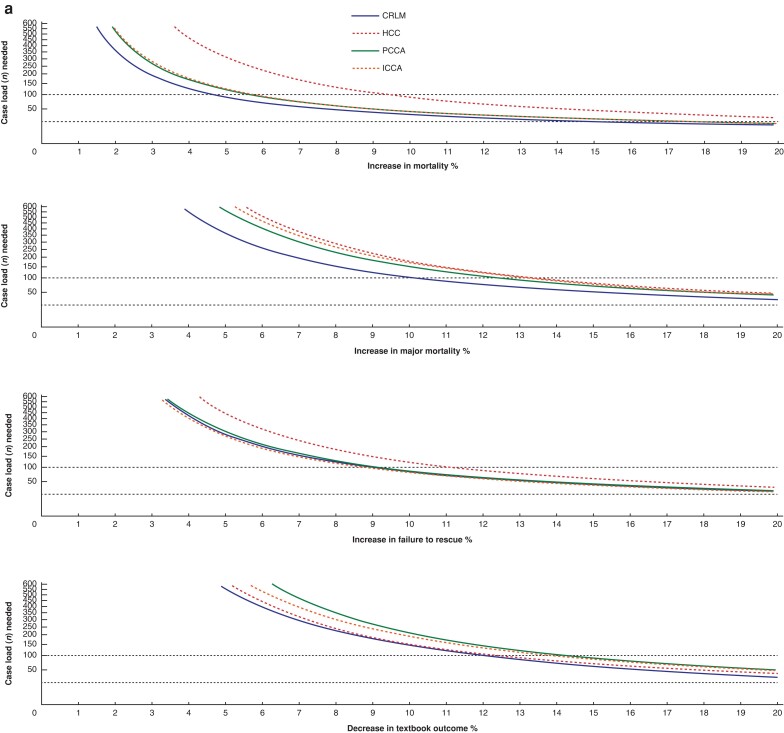

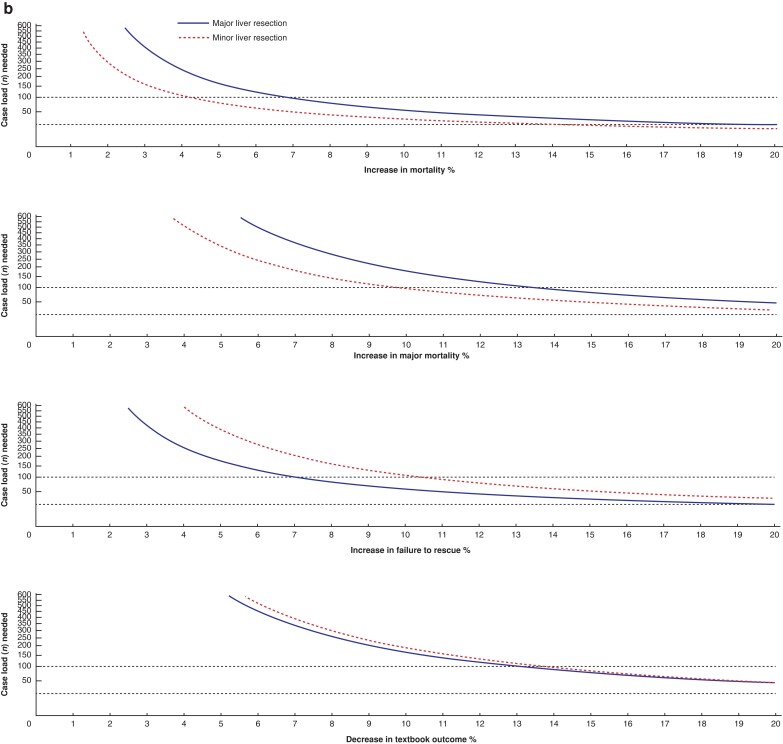

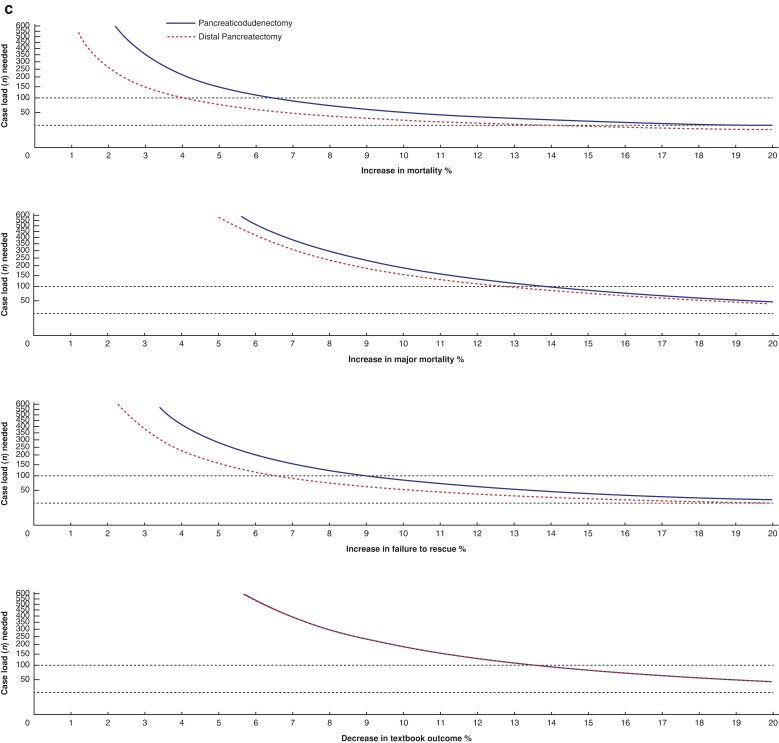
Required caseload per percentage point increase compared to the benchmark for different treatment groups on mortality rate, failure to rescue, major morbidity rate and textbook outcome or ideal outcome

The minimum annual surgical hospital volume needed to reliably distinguish an absolute 15%-point decrease in TO or IO when compared to the benchmark would require a minimum annual hospital volume of 25 minor LR, 29 major LR, 27 PD and 28 DP when measured over a three-year period (*Fig. 2*, *Table S5*). *Tables S2–5* also show the different volumes needed for specific indications of liver surgery.

## Discussion

This nationwide audit-based study exposed the complexity of benchmarking quality of care in complex, relatively low-frequency surgical procedures in HPB surgery. Four commonly used quality indicators were evaluated. Current event rates and minimum volume requirements per hospital are too low to detect any meaningful between-hospital differences in mortality rate and failure to rescue. Major morbidity rate and textbook or ideal outcome are better candidates to use for benchmarking.

In the current setting, mortality and failure to rescue rates should be interpreted carefully when the wish is to assess hospital differences. It should not lead to the premature conclusion that there are no differences between centres. This is in line with previous studies reporting that mortality rate was not an ideal indicator for benchmarking hospital performance in other surgical specialties, such as paediatric surgery and bariatric surgery^16–19^.

Obviously, collecting data on mortality rate and failure to rescue is not useless. Feedback to individual centres allows for periodic evaluation and monitoring of their results at a local level. FTR is often used as a study endpoint in several trials. In this context FTR can give valuable information^20^. However, when the aim is benchmarking hospital quality in HPB surgery, or the appraisal of quality of care at a national level, single or composite outcome parameters with higher event rates should be selected. TO or IO are better suited for these purposes as more events are merged into one indicator. A meaningful change in these quality indicators can be reliably detected with lower hospital volumes due to a higher event rate. An essential limitation of composite endpoints as quality indicators is that the interpretation is more complex. It is recommended to also show the separate components of these composite indicators to show where improvements can be made.^13^ In addition, ‘benchmarking’ requires a basic organization of the healthcare system. It is also important that there is (inter)national consensus of the definitions used.

Clinical auditing serves as a tool to improve healthcare. This study specifically assessed different volumes to effectively distinguish significant outliers but did not explore volume–outcome relationship. Notably, there is an absence of widely accepted cut-offs to assess outliers and these cut-offs may vary depending on the mean event rate. Therefore, it is important that surgeons, hospitals, policymakers, healthcare insurance companies and patient organizations engage in discussions about clinically relevant detection thresholds. The assessment of outlier status will vary across indicators. A mortality rate 5% higher than the benchmark would be too large a difference to indicate a hospital as an outlier from the perspective of the authors, whereas a 10% lower rate in TO may be both relevant and reasonable to distinguish an outlier. Higher volumes allow for detection of more subtle differences and may better define a hospital as an outlier, yet volumes will be excessive (>1800 patients per hospital) if an absolute 1%-point difference should be detected. Consensus on which range of differences between an outlier hospital and the benchmark are clinically relevant must be determined before a minimum annual hospital volume can be recommended.

Increasing annual hospital volume to increase and benchmark quality of care is often mentioned.^21,22^ However, increasing minimum volumes and the challenge of low event rates can be approached in several ways. One could consider both increasing the annual hospital volume and extending the period during which quality is measured. Further concentration to increase hospital volumes obviously has serious consequences for capacity and accessibility. Pooling data of several consecutive years to obtain an adequate volume of procedures per hospital is a straightforward approach without increasing the annual minimum hospital volume with limited effects on capacity and accessibility. Pooling data, however, is questionable because several other changing factors over time could influence outcomes, such as new surgical techniques or guidelines. For example, the indications for liver and pancreatic surgery significantly changed over the last decade and the proportion of minimally invasive resections increased immensely in the last five years.^20,23^ Reporting over more extended time periods may not reflect current quality. Moreover, detection of improved or worsened quality of care is delayed. The authors consider reporting over two to three consecutive years as a reasonable period.

Finally, benchmarking could be performed between clusters of hospitals or regions. This obviously increases volume. However, regional network formation varies immensely across Europe. A prerequisite for comparing networks is to align care pathways preoperatively, intra-operatively and post-surgery, and to have multidisciplinary team meetings.

This study gives a new perspective on quality indicators used in clinical auditing, but the results should be interpreted in light of some limitations. The Netherlands is a small, densely populated country in which auditing was used since 2013 ultimately to improve quality of care. A different population or a different healthcare system may have different benchmarks and higher variability. Further subtyping according to tumour type was not performed, as for most tumour types the total hospital volumes were too low. The authors acknowledge that, for example, a major liver resection for colorectal liver metastases is associated with lower major morbidity and mortality rates than a major liver resection for HCC in a cirrhotic liver. Further subtyping according to surgery was not performed.

Pancreatoduodenectomy for duodenal cancer with a soft pancreas with a small pancreatic duct is likely to have a higher risk of complications than pancreatoduodenectomy for a small, resectable cancer in the pancreatic head with a hard pancreas and wide pancreatic duct. Then, both care pathways for liver and pancreatic resection have some features in common such as the desire to have high-quality interventional radiology when intra-abdominal complications occur. In the current analysis, the synergistic effect of performing both liver and pancreatic surgery in the same hospital was not investigated. The authors defined the mean event rate in 2020 and 2021 as benchmark. Others used data of high-volume centres only^24^. Varying the benchmark may influence outcomes. Stakeholders should agree in their discussions on what the benchmark is. Finally, the significance of considering case-mix differences among hospitals is recognized. In this study, however, concerns regarding sample size adequacy were prioritized. Without sufficient sample size, even impeccable risk adjustment may yield inconclusive results.

## Supplementary Material

znae119_Supplementary_Data

## Data Availability

Upon request.
