# Supplementary material for: Assessing quality of hepato-pancreato-biliary surgery: nationwide benchmarking
Source: Br J Surg. 2024 May 15;111(5):znae119. doi: 10.1093/bjs/znae119 (PMC11095128; doi:10.1093/bjs/znae119)
Supplement: znae119_Supplementary_Data [file znae119_supplementary_data.docx]

**Assessing quality of hepato-pancreato-biliary surgery: nationwide benchmarking**

Michelle R. de Graaff MD^1,2*^, Tessa E. Hendriks MD^1,3,4,5*^, Michel Wouters MD PhD^1,6,7^, Mark Nielen PhD^1^, Ignace de Hingh MD PhD^8^, Bas Groot Koerkamp MD PhD9, I. Hjalmar C. van Santvoort MD PhD^10.11^, Olivier R. Busch MD, PhD^3,4^, Marcel den Dulk MD PhD^12,13^, Joost M. Klaase MD PhD^2^, Erik van Zwet PhD^7^, Bert A. Bonsing MD PhD^5^, Dirk J. Grünhagen MD PhD^9^, Marc G. Besselink MD, PhD^3,4#^, Niels F.M. Kok MD PhD^6#^ for the Dutch Hepato Biliary Audit (DHBA), Dutch Pancreatic Cancer audit (DPCA), and the Dutch Institute of Clinical Auditing (DICA)

^*^Shared first authorship, ^#^shared senior authorship

1. Dutch Institute for Clinical Auditing, Scientific Bureau, Leiden, the Netherlands
2. Department of Hepato-Pancreato-Biliary Surgery and Liver Transplantation, University Medical Centre Groningen, Groningen, the Netherlands
3. Amsterdam UMC, location University of Amsterdam, Department of Surgery, Amsterdam, the Netherlands
4. Cancer Centre Amsterdam, the Netherlands
5. Department of Surgery, Leiden University Medical Centre, Leiden, the Netherlands
6. Department of Surgery, Antoni van Leeuwenhoek – Dutch Cancer Institute, Amsterdam, the Netherlands
7. Department of Biomedical Data Sciences, Leiden University Medical Centre
8. Department of Surgery, Catharina Hospital, Eindhoven, the Netherlands
9. Department of Surgery, Erasmus MC Cancer Institute, Rotterdam, the Netherlands
10. Department of Surgery, University Medical Centre Utrecht, Utrecht, the Netherlands
11. Department of Surgery, St Antonius Hospital, Nieuwegein, the Netherlands
12. Department of Surgery, Maastricht University Medical Centre, Maastricht, the Netherlands
13. NUTRIM-School of Nutrition and Translational Research in Metabolism, Maastricht University, Maastricht, The Netherlands.

Funding: None

Category manuscript: original article

**Corresponding author:**

Tessa E. Hendriks, MD

Department of Surgery, Amsterdam University Medical Center

De Boelelaan 1117 (ZH-7F)

1081 HV Amsterdam the Netherlands

+3120444400

Email: [t.hendriks@dica.nl](mailto:t.hendriks@dica.nl)

**ORCID ID** 0009-0007-7418-3056; **Twitter** @TEHendriks

**Index**

[Supplementary tables and figures 3](#_Toc160632904)

[Supplementary Table 1 3](#_Toc160632905)

[Supplementary Figure 1 3](#_Toc160632906)

[Supplementary Table 2 Mortality 3](#_Toc160632907)

[Supplementary Table 3 Failure to rescue (FTR) 3](#_Toc160632908)

[Supplementary Table 4 Major Morbidity 3](#_Toc160632909)

[Supplementary Table 5 Ideal Outcome and Textbook Outcome 3](#_Toc160632910)

[References 10](#_Toc160632911)

# Supplementary tables and figures

Supplementary Table 1 Number of hospitals performing specific procedures and nationwide average rates (benchmark) of quality indicators, all numbers are calculated with data from 2020 and 2021.

Supplementary Figure 1**.** Sample size proportion test equation.

## Supplementary Table 2 Mortality

Number of hospitals that exceed the minimal caseload for varying number of years and varying thresholds of differences compared to the current mortality benchmark.

**Two-fold indicates 2 times the current benchmark.* ******** *For example, in case of minor liver resections to detect a 2%-point difference compared to the current benchmark, a hospital volume of 275 is required. With current volumes, only 6 hospitals met this required volume when pooled over 5 consecutive years.*

## Supplementary Table 3 Failure to rescue (FTR)

Number of hospitals that exceed the minimal caseload for varying number of years and varying thresholds of differences compared to the current failure to rescue (FTR) benchmark.

**Two-fold indicates 2 times the current benchmark.*

## Supplementary Table 4 Major Morbidity

Number of hospitals that exceed the minimal caseload for varying number of years and varying thresholds of differences compared to the current major morbidity benchmark.

**Two-fold indicates 2 times the current benchmark.*

## Supplementary Table 5 Ideal Outcome and Textbook Outcome

Number of hospitals that exceed the minimal caseload for varying number of years and varying thresholds of differences compared to the current ideal outcome (IO) and Textbook Outcome (TO) benchmark.

**Two-fold indicates 2 times the current benchmark.*

**Supplementary Table 1** Number of hospitals performing specific procedures and nationwide average rates (benchmark) of quality indicators, all numbers are calculated with data from 2020 and 2021.

|  | **Liver resection** | | | |
| --- | --- | --- | --- | --- |
|  | **CRLM** | **HCC** | **pCCC** | **iCCC** |
| Number of hospitals | 22 | 13 | 6 | 13 |
| Median number of procedures per hospital (min-max) in 2021 | 36.5 (10 – 73) | 7 (1 – 23) | 8 (1– 16) | 3 (1 -10) |
| Nationwide average mortality (min- max) in % | 1.0 (0 – 3.2) | 1.8 (0 – 12.5) | 8.8 (0 – 100) | 2.0 (0 – 16.7) |
| Nationwide average major morbidity (min- max) in % | 11.6 (3.6 – 32.3) | 21.3 (0 – 40) | 58.8 (0 – 66.7) | 28.0 (0 – 50) |
| Nationwide average failure to rescue (min- max) in % | 7.7 (0 – 25) | 8.3 (0 – 66.7) | 14.9 (0 – 100) | 7.1 (0 – 100) |
| Nationwide average textbook outcome (min- max) in % | 79 (61.3 – 94.4) | 71.6 (0 – 100) | 31.2 (0 – 57.1) | 60.0 (25 – 100) |

**Supplementary Figure 1.** Sample size proportion test equation.


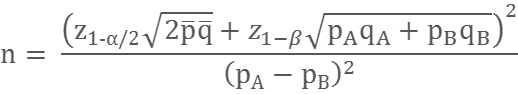


**Supplementary Table 2 Mortality**

*Number of hospitals that exceed the minimal caseload for varying number of years and varying thresholds of differences compared to the current mortality benchmark.*

| ***Surgical Procedure*** | ***Benchmark*** | ***Absolute %-point increase over benchmark*** | ***Threshold volume needed*** | ***Hospitals meeting or exceeding required volume threshold (N)%*** | | | | |
| --- | --- | --- | --- | --- | --- | --- | --- | --- |
|  |  |  |  | ***1 year*** | ***2 years*** | ***3 years*** | ***5 years*** | ***8 years*** |
| **Liver resection** |  |  |  |  |  |  |  |  |
| Minor liver resection | 0.6% | Two-fold* | 1889 | 0/21 | 0/21 | 0/21 | 0/21 | 0/21 |
|  |  | 1% | 808 | 0/21 | 0/21 | 0/21 | 0/21 | 0/21 |
|  |  | 2% | 275 | 0/21 | 0/21 | 0/21 | 6/21 | 13/21 |
|  |  | 5% | 75 | 3/21 | 14/21 | 17/21 | 21/21 | 21/21 |
|  |  | 10% | 31 | 15/21 | 20/21 | 21/21 | 21/21 | 21/21 |
| Major liver resection | 3.3% | Two-fold* | 237 | 0/21 | 0/21 | 0/21 | 0/21 | 4/21 |
|  |  | 1% | 2857 | 0/21 | 0/21 | 0/21 | 0/21 | 0/21 |
|  |  | 2% | 797 | 0/21 | 0/21 | 0/21 | 0/21 | 0/21 |
|  |  | 5% | 164 | 0/21 | 0/21 | 0/21 | 4/21 | 6/21 |
|  |  | 10% | 54 | 1/21 | 4/21 | 6/21 | 11/21 | 15/21 |
| **Pancreatic resection** | |  |  |  |  |  |  |  |
| Pancreaticoduodenectomy | 2.7% | Two-fold* | 407 | 0/15 | 0/15 | 0/14 | 2/14 | 3/14 |
|  |  | 1% | 2417 | 0/15 | 0/15 | 0/14 | 0/14 | 0/14 |
|  |  | 2% | 686 | 0/15 | 0/15 | 0/14 | 0/15 | 2/14 |
|  |  | 5% | 146 | 0/15 | 3/15 | 5/14 | 11/14 | 14/14 |
|  |  | 10% | 49 | 7/15 | 12/15 | 14/14 | 14/14 | 14/14 |
| Distal pancreatectomy | 0.6% | Two-fold* | 1889 | 0/15 | 0/15 | 0/14 | 0/14 | 0/14 |
|  |  | 1% | 808 | 0/15 | 0/15 | 0/14 | 0/14 | 0/14 |
|  |  | 2% | 275 | 0/15 | 0/15 | 0/14 | 0/14 | 0/14 |
|  |  | 5% | 75 | 0/15 | 0/15 | 3/14 | 4/14 | 9/14 |
|  |  | 10% | 31 | 0/15 | 3/15 | 9/14 | 13/14 | 14/14 |
| **Liver surgery** |  |  |  |  |  |  |  |  |
| CRLM | 1.0% | Two-fold* | 1126 | 0/21 | 0/21 | 0/21 | 0/21 | 0/21 |
|  |  | 1% | 1126 | 0/21 | 0/21 | 0/21 | 0/21 | 0/21 |
|  |  | 5% | 90 | 0/21 | 8/21 | 15/21 | 19/21 | 21/21 |
|  |  | 10% | 35 | 12/21 | 19/21 | 21/21 | 21/21 | 21/21 |
| HCC | 1.8% | Two-fold* | 619 | 0/12 | 0/12 | 0/12 | 0/12 | 0/12 |
|  |  | 1% | 1742 | 0/12 | 0/12 | 0/12 | 0/12 | 0/12 |
|  |  | 5% | 117 | 0/12 | 0/12 | 0/12 | 1/12 | 3/12 |
|  |  | 10% | 42 | 0/12 | 1/12 | 4/12 | 6/12 | 7/12 |
| pCCA | 8.8% | Two-fold* | 113 | 0/6 | 0/6 | 0/6 | 0/6 | x |
|  |  | 1% | 6616 | 0/6 | 0/6 | 0/6 | 0/6 | x |
|  |  | 5% | 311 | 0/6 | 0/6 | 0/6 | 0/6 | x |
|  |  | 10% | 90 | 0/6 | 0/6 | 0/6 | 0/6 | x |
| iCCA | 2.0% | Two-fold* | 555 | 0/12 | 0/12 | 0/12 | 0/12 | x |
|  |  | 1% | 1894 | 0/12 | 0/12 | 0/12 | 0/12 | x |
|  |  | 5% | 124 | 0/12 | 0/12 | 0/12 | 0/12 | x |
|  |  | 10% | 44 | 0/12 | 0/12 | 0/12 | 1/12 | x |

**Two-fold indicates 2 times the current benchmark.* ******** *For example, in case of minor liver resections to detect a 2%-point difference compared to the current benchmark, a hospital volume of 275 is required. With current volumes, only 6 hospitals met this required volume when pooled over 5 consecutive years.*

**Supplementary Table 3 Failure to rescue (FTR)**

*Number of hospitals that exceed the minimal caseload for varying number of years and varying thresholds of differences compared to the current failure to rescue (FTR) benchmark.*

| ***Surgical Procedure*** | ***Benchmark*** | ***Absolute %-point increase over benchmark*** | ***Threshold volume needed*** | ***Hospitals meeting or exceeding required volume threshold (N)%*** | | | | |
| --- | --- | --- | --- | --- | --- | --- | --- | --- |
|  |  |  |  | ***1 year*** | ***2 years*** | ***3 years*** | ***5 years*** | ***8 years*** |
| **Liver resection** |  |  |  |  |  |  |  |  |
| Minor liver resection | 5.4% | Two-fold* | 195 | 0/21 | 0/21 | 5/21 | 11/21 | 18/21 |
|  |  | 1% | 4350 | 0/21 | 0/21 | 0/21 | 0/21 | 0/21 |
|  |  | 2% | 1169 | 0/21 | 0/21 | 0/21 | 0/21 | 0/21 |
|  |  | 5% | 223 | 0/21 | 0/21 | 5/21 | 12/21 | 18/21 |
|  |  | 10% | 69 | 4/21 | 14/21 | 18/21 | 21/21 | 21/21 |
| Major liver resection | 14.2% | Two-fold* | 64 | 0/21 | 4/21 | 6/21 | 10/21 | 14/21 |
|  |  | 1% | 9839 | 0/21 | 0/21 | 0/21 | 0/21 | 0/21 |
|  |  | 2% | 2527 | 0/21 | 0/21 | 0/21 | 0/21 | 0/21 |
|  |  | 5% | 435 | 0/21 | 0/21 | 0/21 | 0/21 | 0/21 |
|  |  | 10% | 120 | 0/21 | 0/21 | 2/21 | 6/21 | 8/21 |
| **Pancreatic resection** | |  |  |  |  |  |  |  |
| Pancreaticoduodenectomy | 7.5% | Two-fold* | 136 | 1/15 | 4/15 | 5/14 | 12/14 | 14/14 |
|  |  | 1% | 5771 | 0/15 | 0/15 | 0/14 | 0/14 | 0/14 |
|  |  | 2% | 1521 | 0/15 | 0/15 | 0/14 | 0/14 | 0/14 |
|  |  | 5% | 278 | 0/15 | 1/15 | 2/14 | 3/14 | 8/14 |
|  |  | 10% | 83 | 2/15 | 7/15 | 11/14 | 14/14 | 14/14 |
| Distal pancreatectomy | 3.1% | Two-fold* | 352 | 0/15 | 0/15 | 0/14 | 0/14 | 0/14 |
|  |  | 1% | 2711 | 0/15 | 0/15 | 0/14 | 0/14 | 0/14 |
|  |  | 2% | 760 | 0/15 | 0/15 | 0/14 | 0/14 | 0/14 |
|  |  | 5% | 158 | 0/15 | 0/15 | 0/14 | 1/14 | 3/14 |
|  |  | 10% | 52 | 0/15 | 3/15 | 3/14 | 8/14 | 13/14 |
| **Liver surgery** |  |  |  |  |  |  |  |  |
| CRLM | 7.7% | Two-fold* | 132 | 0/21 | 3/21 | 9/21 | 17/21 | 20/21 |
|  |  | 1% | 5903 | 0/21 | 0/21 | 0/21 | 0/21 | 0/21 |
|  |  | 2% | 1554 | 0/21 | 0/21 | 0/21 | 0/21 | 0/21 |
|  |  | 5% | 283 | 0/21 | 0/21 | 3/21 | 6/21 | 12/21 |
|  |  | 10% | 84 | 0/21 | 9/21 | 15/21 | 19/21 | 21/21 |
| HCC | 8.3% | Two-fold* | 121 | 0/12 | 0/12 | 0/12 | 1/12 | 3/12 |
|  |  | 1% | 6294 | 0/12 | 0/12 | 0/12 | 0/12 | 0/12 |
|  |  | 2% | 1651 | 0/12 | 0/12 | 0/12 | 0/12 | 0/12 |
|  |  | 5% | 299 | 0/12 | 0/12 | 0/12 | 0/12 | 0/12 |
|  |  | 10% | 87 | 0/12 | 0/12 | 0/12 | 1/12 | 6/12 |
| pCCA | 14.9% | Two-fold* | 60 | 0/6 | 0/6 | 0/6 | 0/6 | x |
|  |  | 1% | 10223 | 0/6 | 0/6 | 0/6 | 0/6 | x |
|  |  | 2% | 2621 | 0/6 | 0/6 | 0/6 | 0/6 | x |
|  |  | 5% | 449 | 0/6 | 0/6 | 0/6 | 0/6 | x |
|  |  | 10% | 123 | 0/6 | 0/6 | 0/6 | 0/6 | x |
| iCCA | 7.1% | Two-fold* | 144 | 0/12 | 0/12 | 0/12 | 0/12 | x |
|  |  | 1% | 5506 | 0/12 | 0/12 | 0/12 | 0/12 | x |
|  |  | 2% | 1455 | 0/12 | 0/12 | 0/12 | 0/12 | x |
|  |  | 5% | 268 | 0/12 | 0/12 | 0/12 | 0/12 | x |
|  |  | 10% | 80 | 0/12 | 0/12 | 0/12 | 0/12 | x |

**Two-fold indicates 2 times the current benchmark.*

**Supplementary Table 4 Major Morbidity**

*Number of hospitals that exceed the minimal caseload for varying number of years and varying thresholds of differences compared to the current major morbidity benchmark.*

| ***Surgical Procedure*** | ***Benchmark*** | ***Absolute %-point increase over benchmark*** | ***Threshold volume needed*** | ***Hospitals meeting or exceeding required volume threshold (N)%*** | | | | |
| --- | --- | --- | --- | --- | --- | --- | --- | --- |
|  |  |  |  | ***1 year*** | ***2 years*** | ***3 years*** | ***5 years*** | ***8 years*** |
| **Liver resection** |  |  |  |  |  |  |  |  |
| Minor liver resection | 9.8% | Two-fold* | 100 | 0/21 | 8/21 | 14/21 | 19/21 | 21/21 |
|  |  | 5% | 335 | 0/21 | 0/21 | 0/21 | 4/21 | 11/21 |
|  |  | 10% | 96 | 0/21 | 9/21 | 14/21 | 18/21 | 21/21 |
|  |  | 15% | 47 | 9/21 | 17/21 | 20/21 | 21/21 | 21/21 |
|  |  | 25% | 20 | 17/21 | 20/21 | 21/21 | 21/21 | 21/21 |
| Major liver resection | 28.1% | Two-fold* | 24 | 5/21 | 10/21 | 13/21 | 16/21 | 20/21 |
|  |  | 5% | 666 | 0/21 | 0/21 | 0/21 | 0/21 | 0/21 |
|  |  | 10% | 173 | 0/21 | 0/21 | 0/21 | 4/21 | 5/21 |
|  |  | 15% | 79 | 0/21 | 3/21 | 4/21 | 6/21 | 10/21 |
|  |  | 25% | 30 | 4/21 | 8/21 | 11/21 | 18/21 | 20/21 |
| **Pancreatic resection** | |  |  |  |  |  |  |  |
| Pancreaticoduodenectomy | 36.0% | Two-fold* | 14 | 15/15 | 15/15 | 14/14 | 14/14 | 14/14 |
|  |  | 5% | 743 | 0/15 | 0/15 | 0/14 | 0/14 | 1/14 |
|  |  | 10% | 189 | 0/15 | 2/15 | 4/14 | 9/14 | 12/14 |
|  |  | 15% | 85 | 2/15 | 6/15 | 10/14 | 14/14 | 14/14 |
|  |  | 25% | 31 | 12/15 | 15/15 | 14/14 | 14/14 | 14/14 |
| Distal pancreatectomy | 22.3% | Two-fold* | 34 | 0/15 | 3/15 | 8/14 | 13/14 | 14/14 |
|  |  | 5% | 584 | 0/15 | 0/15 | 0/14 | 0/14 | 0/14 |
|  |  | 10% | 155 | 0/15 | 0/15 | 0/14 | 0/14 | 0/14 |
|  |  | 15% | 72 | 0/15 | 1/15 | 3/14 | 4/14 | 10/14 |
|  |  | 25% | 28 | 3/15 | 4/15 | 10/14 | 14/14 | 14/14 |
| **Liver surgery** |  |  |  |  |  |  |  |  |
| CRLM | 11.6% | Two-fold* | 82 | 0/21 | 10/21 | 15/21 | 18/21 | 21/21 |
|  |  | 5% | 377 | 0/21 | 0/21 | 0/21 | 0/21 | 8/21 |
|  |  | 10% | 106 | 0/21 | 4/21 | 12/21 | 17/21 | 21/21 |
|  |  | 15% | 52 | 7/21 | 15/21 | 18/21 | 21/21 | 21/21 |
|  |  | 25% | 21 | 17/21 | 20/21 | 21/21 | 21/21 | 21/21 |
| HCC | 21.3% | Two-fold* | 37 | 0/12 | 1/12 | 4/12 | 7/12 | 7/12 |
|  |  | 5% | 568 | 0/12 | 0/12 | 0/12 | 0/12 | 0/12 |
|  |  | 10% | 151 | 0/12 | 0/12 | 0/12 | 0/12 | 2/12 |
|  |  | 15% | 70 | 0/12 | 0/12 | 1/12 | 3/12 | 6/12 |
|  |  | 25% | 27 | 0/12 | 3/12 | 6/12 | 7/12 | 8/12 |
| pCCA | 58.8% | 5% | 744 | 0/6 | 0/6 | 0/6 | 0/6 | x |
|  |  | 10% | 180 | 0/6 | 0/6 | 0/6 | 0/6 | x |
|  |  | 15% | 77 | 0/6 | 0/6 | 0/6 | 0/6 | x |
|  |  | 25% | 25 | 0/6 | 0/6 | 3/6 | 4/6 | x |
| iCCA | 28% | Two-fold* | 24 | 0/12 | 0/12 | 2/12 | 3/12 | x |
|  |  | 5% | 664 | 0/12 | 0/12 | 0/12 | 0/12 | x |
|  |  | 10% | 173 | 0/12 | 0/12 | 0/12 | 0/12 | x |
|  |  | 15% | 79 | 0/12 | 0/12 | 0/12 | 0/12 | x |
|  |  | 25% | 29 | 0/12 | 0/12 | 0/12 | 2/12 | x |

**Two-fold indicates 2 times the current benchmark.*

**Supplementary Table 5 Ideal Outcome and Textbook Outcome**

*Number of hospitals that exceed the minimal caseload for varying number of years and varying thresholds of differences compared to the current ideal outcome (IO) and Textbook Outcome (TO) benchmark.*

| ***Surgical Procedure*** | ***Benchmark*** | ***Absolute %-point decrease to benchmark*** | ***Threshold volume needed*** | ***Hospitals meeting or exceeding required volume threshold (N)%*** | | | | |
| --- | --- | --- | --- | --- | --- | --- | --- | --- |
|  |  |  |  | ***1 year*** | ***2 years*** | ***3 years*** | ***5 years*** | ***8 years*** |
| **Liver resection** |  |  |  |  |  |  |  |  |
| Minor liver resection | 73.6% | 5% | 664 | 0/21 | 0/21 | 0/21 | 0/21 | 3/21 |
|  |  | 10% | 168 | 0/21 | 1/21 | 5/21 | 14/21 | 18/21 |
|  |  | 15% | 77 | 3/21 | 13/21 | 16/21 | 18/21 | 21/21 |
|  |  | 25% | 29 | 15/21 | 20/21 | 21/21 | 21/21 | 21/21 |
| Major liver resection | 54.1% | 5% | 783 | 0/21 | 0/21 | 0/21 | 0/21 | 0/21 |
|  |  | 10% | 196 | 0/21 | 0/21 | 0/21 | 3/21 | 4/21 |
|  |  | 15% | 86 | 0/21 | 1/21 | 4/21 | 7/21 | 10/21 |
|  |  | 25% | 30 | 4/21 | 8/21 | 11/21 | 16/21 | 19/21 |
| **Pancreatic resection** | |  |  |  |  |  |  |  |
| Pancreaticoduodenectomy | 46.8% | 5% | 774 | 0/15 | 0/15 | 0/14 | 0/14 | 1/14 |
|  |  | 10% | 190 | 0/15 | 2/15 | 4/14 | 9/14 | 2/14 |
|  |  | 15% | 82 | 2/15 | 7/15 | 12/14 | 14/14 | 14/14 |
|  |  | 25% | 27 | 13/15 | 15/15 | 14/14 | 14/14 | 14/14 |
| Distal pancreatectomy | 63.3% | 5% | 748 | 0/15 | 0/15 | 0/14 | 0/14 | 0/14 |
|  |  | 10% | 190 | 0/15 | 0/15 | 0/14 | 0/14 | 3/14 |
|  |  | 15% | 85 | 0/15 | 0/15 | 2/14 | 3/14 | 7/14 |
|  |  | 25% | 31 | 0/15 | 3/15 | 9/14 | 13/14 | 14/14 |
| **Liver surgery** |  |  |  |  |  |  |  |  |
| CRLM | 79% | 5% | 443 | 0/21 | 0/21 | 0/21 | 0/21 | 6/21 |
|  |  | 10% | 118 | 0/21 | 4/21 | 11/21 | 17/21 | 21/21 |
|  |  | 15% | 55 | 5/21 | 15/21 | 18/21 | 21/21 | 21/21 |
|  |  | 25% | 21 | 18/21 | 20/21 | 21/21 | 21/21 | 21/21 |
| HCC | 71.6% | 5% | 669 | 0/12 | 0/12 | 0/12 | 0/12 | 0/12 |
|  |  | 10% | 174 | 0/12 | 0/12 | 0/12 | 0/12 | 1/12 |
|  |  | 15% | 79 | 0/12 | 0/12 | 0/12 | 3/12 | 6/12 |
|  |  | 25% | 30 | 0/12 | 3/12 | 6/12 | 7/12 | 8/12 |
| pCCA | 31.2% | 5% | 641 | 0/6 | 0/6 | 0/6 | 0/6 | x |
|  |  | 10% | 151 | 0/6 | 0/6 | 0/6 | 0/6 | x |
|  |  | 15% | 61 | 0/6 | 0/6 | 0/6 | 0/6 | x |
|  |  | 25% | 17 | 0/6 | 2/6 | 4/6 | 4/6 | x |
| iCCA | 60% | 5% | 767 | 0/12 | 0/12 | 0/12 | 0/12 | x |
|  |  | 10% | 194 | 0/12 | 0/12 | 0/12 | 0/12 | x |
|  |  | 15% | 86 | 0/12 | 0/12 | 0/12 | 0/12 | x |
|  |  | 25% | 31 | 0/12 | 0/12 | 0/12 | 1/12 | x |

**Two-fold indicates 2 times the current benchmark.*

# References

1 Donabedian A. The quality of care. How can it be assed? *JAMA*. 1988; **260**: 1743–1748.

2 Beck N, van Bommel AC, Eddes EH, van Leersum NJ, Tollenaar RA, Wouters MW, *et al.* The Dutch Institute for Clinical Auditing: Achieving Codman’s Dream on a Nationwide Basis. *Ann Surg* [Internet]. 2020; **271**. Available from: https://journals.lww.com/annalsofsurgery/Fulltext/2020/04000/The_Dutch_Institute_for_Clinical_Auditing_.7.aspx

3 Berg M, Meijerink Y, Gras M, Goossensen A, Schellekens W, Haeck J, *et al.* Feasibility first: Developing public performance indicators on patient safety and clinical effectiveness for Dutch hospitals. *Health Policy (New York)*. Elsevier; 2005 Dec 1; **75**: 59–73.

4 Elfrink AKEE, Kok NFMM, Swijnenburg R-JJ, den Dulk M, van den Boezem PB, Hartgrink HH, *et al.* Nationwide oncological networks for resection of colorectal liver metastases in the Netherlands: Differences and postoperative outcomes. *European Journal of Surgical Oncology*. Eur J Surg Oncol; 2022 Sep 1; **48**: 435–448.

5 Suurmeijer JA, Henry AC, Bonsing BA, Bosscha K, van Dam RM, van Eijck CH, *et al.* Outcome of Pancreatic Surgery During the First Six Years of a Mandatory Audit  within the Dutch Pancreatic Cancer Group. *Ann Surg*. United States; 2022 Jul;

6 Ministerie van Volksgezondheid en Welzijn. Ontwikkeling Uitkomstgerichte zorg 2018-2022. [cited 2023 Apr 26]; Available from: https://open.overheid.nl/documenten/ronl-b0848781-3c90-4b03-9515-f6b6a4cc168e/pdf

7 Zorginstituut Nederland. Passende zorg in oncologieregio’s: plan van aanpak netwerk- en expertzorg voor mensen met kanker [Internet]. 2023 [cited 2023 Aug 16]. Available from: https://www.zorginstituutnederland.nl/binaries/zinl/documenten/rapport/2023/06/29/passende-zorg-in-oncologieregios-plan-van-aanpak-netwerk--en-expertzorg-voor-mensen-met-kanker/Plan+van+aanpak+Passende+zorg+in+oncologieregio%27s.pdf

8 van Rijssen LB, Koerkamp BG, Zwart MJ, Bonsing BA, Bosscha K, van Dam RM, *et al.* Nationwide prospective audit of pancreatic surgery: design, accuracy, and outcomes of the Dutch Pancreatic Cancer Audit. *HPB*. Elsevier; 2017 Oct 1; **19**: 919–926.

9 van der Werf LR, Voeten SC, van Loe CMM, Karthaus EG, Wouters MWJM, Prins HA. Data verification of nationwide clinical quality registries. *BJS Open*. NLM (Medline); 2019 Dec 1; **3**: 857–864.

10 Olthof PB, Elfrink AKE, Marra E, Belt EJT, van den Boezem PB, Bosscha K, *et al.* Volume–outcome relationship of liver surgery: a nationwide analysis. *British Journal of Surgery*. John Wiley and Sons Ltd; 2020 Jun 1; **107**: 917–926.

11 Multidisciplinaire normering oncologische zorg in Nederland Platform Oncologie-SONCOS. [cited 2023 Aug 14]; Available from: https://demedischspecialist.nl/sites/default/files/SONCOS-normeringsrapport-versie-9-2021.pdf

12 Strijker M, Mackay TM, Bonsing BA, Bruno MJ, Van Eijck CHJ, De Hingh IHJT, *et al.* Establishing and Coordinating a Nationwide Multidisciplinary Study Group: Lessons Learned by the Dutch Pancreatic Cancer Group. *Ann Surg*. 2020; **271**: E102–E104.

13 de Graaff MR, Elfrink AKE, Buis CI, Swijnenburg RJ, Erdmann JI, Kazemier G, *et al.* Defining Textbook Outcome in liver surgery and assessment of hospital variation: A nationwide population-based study. *European Journal of Surgical Oncology*. W.B. Saunders; 2022 Jun 20;

14 Augustinus S, Mackay TM, Andersson B, Beane JD, Busch OR, Gleeson EM, *et al.* Ideal Outcome After Pancreatoduodenectomy. *Ann Surg* [Internet]. 2023 Jul 21 [cited 2023 Aug 14]; Available from: https://journals.lww.com/annalsofsurgery/fulltext/9900/ideal_outcome_after_pancreatoduodenectomy__a.582.aspx

15 Bassi C, Marchegiani G, Dervenis C, Sarr M, Abu Hilal M, Adham M, *et al.* The 2016 update of the International Study Group (ISGPS) definition and grading  of postoperative pancreatic fistula: 11 Years After. *Surgery*. United States; 2017 Mar; **161**: 584–591.

16 Dimick JB, Welch HG, Birkmeyer JD, Gilbert Welch H, Birkmeyer JD. Surgical Mortality as an Indicator of Hospital Quality: The Problem With Small Sample Size. *JAMA*. American Medical Association; 2004 Aug 18; **292**: 847–851.

17 Bardach NS, Chien AT, Dudley RA. Small Numbers Limit the Use of the Inpatient Pediatric Quality Indicators for Hospital Comparison. *Acad Pediatr*. Elsevier; 2010 Jul 1; **10**: 266–273.

18 Welke KF, Karamlou T, Ungerleider RM, Diggs BS. Mortality Rate Is Not a Valid Indicator of Quality Differences Between Pediatric Cardiac Surgical Programs. *Ann Thorac Surg*. Elsevier; 2010 Jan 1; **89**: 139–146.

19 Krell RW, Finks JF, English WJ, Dimick JB. Profiling Hospitals on Bariatric Surgery Quality: Which Outcomes Are Most Reliable? *J Am Coll Surg*. NIH Public Access; 2014 Oct 1; **219**: 725.

20 Smits FJ, Henry AC, Besselink MG, Busch OR, van Eijck CH, Arntz M, *et al.* Algorithm-based care versus usual care for the early recognition and management  of complications after pancreatic resection in the Netherlands: an open-label, nationwide, stepped-wedge cluster-randomised trial. *Lancet*. England; 2022 May; **399**: 1867–1875.

21 Khuri SF, Henderson WG. The case against volume as a measure of quality of surgical care. *World J Surg* [Internet]. Springer; 2005 Oct 15 [cited 2023 Sep 4]; **29**: 1222–1229. Available from: https://link.springer.com/article/10.1007/s00268-005-7987-6

22 Dimick JB, Pronovost PJ, Cowan JA, Lipsett PA. Surgical volume and quality of care for esophageal resection: do high-volume hospitals have fewer complications? *Ann Thorac Surg*. Elsevier; 2003 Feb 1; **75**: 337–341.

23 Smits FJ, Henry AC, Besselink MG, Busch OR, van Eijck CH, Arntz M, *et al.* Algorithm-based care versus usual care for the early recognition and management of complications after pancreatic resection in the Netherlands: an open-label, nationwide, stepped-wedge cluster-randomised trial. *Lancet* [Internet]. Lancet; 2022 May 14 [cited 2023 Aug 31]; **399**: 1867–1875. Available from: https://pubmed.ncbi.nlm.nih.gov/35490691/

24 de Graaff MR, Klaase JM, de Kleine R, Elfrink AKE, Swijnenburg R-J, M. Zonderhuis B, *et al.* Practice variation and outcomes of minimally invasive minor liver resections in patients with colorectal liver metastases: a population-based study. *Surgical Endoscopy 2023* [Internet]. Springer; 2023 Apr 18 [cited 2023 Apr 19]; 1–15. Available from: https://link.springer.com/article/10.1007/s00464-023-10010-3

25 Sousa Da Silva RX, Breuer E, Shankar S, Kawakatsu S, Hołówko W, Coelho JS, *et al.* Novel Benchmark Values for Open Major Anatomic Liver Resection in Non-Cirrhotic Patients. A Multicentric Study of 44 International Expert Centers. *Ann Surg*. 2023;
